# Secondary Chromosomal Attachment Site and Tandem Integration of the Mobilizable *Salmonella* Genomic Island 1

**DOI:** 10.1371/journal.pone.0002060

**Published:** 2008-04-30

**Authors:** Benoît Doublet, George R. Golding, Michael R. Mulvey, Axel Cloeckaert

**Affiliations:** 1 INRA, UR1282, Infectiologie Animale et Santé Publique, Nouzilly, France; 2 National Microbiology Laboratory, Public Health Agency of Canada, Winnipeg, Manitoba, Canada; Pasteur Institute, France

## Abstract

**Background:**

The *Salmonella* genomic island 1 is an integrative mobilizable element (IME) originally identified in epidemic multidrug-resistant *Salmonella enterica* serovar Typhimurium (*S*. Typhimurium) DT104. SGI1 contains a complex integron, which confers various multidrug resistance phenotypes due to its genetic plasticity. Previous studies have shown that SGI1 integrates site-specifically into the *S. enterica*, *Escherichia coli*, or *Proteus mirabilis* chromosome at the 3′ end of *thdF* gene (*attB* site).

**Methodology/Principal Findings:**

Here, we report the transfer of SGI1 to a Δ*thdF* mutant of *S.* Typhimurium LT2. In the absence of *thdF*, the frequency of transconjugant formation was reduced by around thirty times of magnitude. Through DNA sequencing SGI1 was shown to integrate specifically into a secondary attachment site (2^nd^ *attB*), which is located in the intergenic region between the chromosomal *sodB* and *purR* genes. At this 2^nd^ *attB* site, we found that a significant fraction of SGI1 transconjugants (43% of wild type and 100% of Δ*thdF* mutant) contained tandem SGI1 arrays. Moreover, in wild type *S.* Typhimurium LT2 transconjugants, SGI1 integrated into both attachment sites, i.e., *thdF* and *sodB*-*purR*. The formation of SGI1 tandem arrays occurred in both specific *attB* sites. There was heterogeneity in the size of the SGI1 tandem arrays detected in single transconjugant colonies. Some arrays consisted as far as six SGI1s arranged in tandem. These tandem arrays were shown to persist during serial passages with or without antibiotic selection pressure.

**Conclusions/Significance:**

The ability of integration into two distinct chromosomal sites and tandem array formation of SGI1 could contribute to its spread and persistence. The existence of a secondary attachment site in the *Salmonella* chromosome has potential implications for the mobility of SGI1, which may integrate in other attachment sites of other bacterial pathogens that do not possess the 1^st^ or 2^nd^ specific SGI1 *attB* sites of *Salmonella*.

## Introduction

Genomic islands are large chromosomal regions that have been acquired by horizontal transfer. They are present in certain bacteria but are absent in most closely related bacteria [1]. Genomic islands often carry genes that bring a selective advantage to the host bacterium in a specific environment. Thus, they are classified into pathogenicity islands which encode virulence determinants, resistance islands which confer multiple antibiotic resistances, xenobiotic degradation islands, and symbiosis islands [1], [2]. They are frequently integrated near or into tRNA genes, flanked by repeat structures, and contain mobility genes coding for integrases or transposases [1]. However, the majority of genomic islands seem to have lost the ability of horizontal transfer. Burrus et al. proposed to classify as integrative and conjugative elements (ICEs), mobile elements which excise from the chromosome by a site-specific recombination, leading to the formation of circular extrachromosomal element; this intermediate is transferred by conjugation and integrates often in a site-specific fashion into the recipient chromosome [3]. The genomic islands are widespread in γ-proteobacteria, however few genomic islands have been characterized as mobile elements [3].

The 43-kb *Salmonella* Genomic island 1 (SGI1) is a *S. enterica*-derived resistance island that was originally identified in epidemic multidrug-resistant *S. enterica* serovar Typhimurium phage type DT104 strains [4], [5]. The SGI1 contains an antibiotic resistance gene cluster conferring resistance to ampicillin (Ap), chloramphenicol (Cm), florfenicol (Ff), streptomycin (Sm), spectinomycin (Sp), sulfonamides (Su), and tetracycline (Tc). The 13-kb SGI1 antibiotic resistance gene cluster is located near the 3′ end of SGI1 and constitutes a complex class 1 integron that belongs to the In4 group, which has been recently named In104 [6], [7]. The In104 integron possesses two cassette attachment sites (*attI1*). At the first *attI1* site of this complex integron, the cassette carries the *aadA2* gene, which confers resistance to Sm and Sp, and downstream a 3′ conserved segment (3′-CS) with a truncated *sul1* gene (*sul1*Δ) is found. At the second *attI1* site, the cassette contains the β-lactamase gene *bla*_PSE-1_ conferring resistance to Ap and downstream the 3′-CS comprises a complete *sul1* gene conferring resistance to Su. Flanked by the two cassettes are the *floR* gene, which confers cross-resistance to Cm and Ff, and the tetracycline resistance genes *tetR* and *tet*(G) . Since the identification of SGI1 in *S.* Typhimurium DT104, variant SGI1 antibiotic resistance gene clusters have been described in a wide variety of *S. enterica* serovars such as Agona, Albany, Cerro, Derby, Dusseldorf, Emek, Infantis, Kentucky, Kiambu, Meleagridis, Newport, and Paratyphi B [5]. Recently, SGI1 and variants of it have been identified in *Proteus mirabilis* clinical and food isolates [5], [8]–[10]. SGI1 variant antibiotic resistance gene clusters were accordingly classified in SGI1-A to SGI1-O [5], [10]–[12]. The identification of SGI1 in *P. mirabilis* clinical isolates is of great concern as the spread of the SGI1 multidrug resistance phenotype could have significant clinical implications in pathogenic bacteria other than *Salmonella*. Potential attachment sites have been identified in diverse human pathogenic bacteria such as *Shigella* spp., *Vibrio* spp., *Pseudomonas* spp., *Brucella* spp., *Legionella pneumophila*, and *Klebsiella pneumoniae* highlighting the potential for SGI1 to emerge in other human pathogens [9].

In 2005, we reported that SGI1 could be conjugally transferred from *S. enterica* donor strains to non-SGI1 *S. enterica* and *Escherichia coli* recipient strains where it integrated into the recipient chromosome in a site-specific manner [13]. Excision of SGI1 from the *Salmonella* chromosome occurs through specific recombination between the 18-bp direct repeats DR-L and DR-R, mediated by the SGI1-encoded integrase gene *int*. After excision, the circular extrachromosomal form of SGI1 harbours a unique 18 bp attachment site (*attP*). After conjugative mobilization in *trans*, the chromosomal integration of SGI1 occurs via a site-specific recombination between the circular form of SGI1 (*attP*) and the specific site at the 3′ end of *thdF* gene (hereafter named primary *attB* site) in the recipient *S. enterica* and *E. coli* chromosome. SGI1 appeared to be a non-self-transmissible but mobilizable element and was thus classified within the group of integrative mobilizable elements (IMEs) that are related to ICEs [13], [14].

In the present study, we report the transfer of SGI1 by conjugation to a *S.* Typhimurium LT2 recipient strain lacking the chromosomal *thdF* gene, i.e. the primary SGI1 *attB* site (1^st^ *attB*). In the absence of *thdF*, we found that SGI1 transfer resulted in the integration of SGI1 in a unique secondary integration site (2^nd^ *attB*) showing conserved regions with the 1^st^ *attB* site. The integration of SGI1 in its 2^nd^ *attB* site always resulted in the formation of extended tandem arrays. These tandem arrays had variable copy numbers of SGI1 in the population of single transconjugants. Our findings suggest that the capacity of multiple site integration and tandem SGI1 arrays may contribute to the spread and persistence of multidrug resistance conferred by SGI1.

## Results

### Conjugative transfer of SGI1 in the absence of the *thdF* gene in *S*. Typhimurium LT2 recipient strain

To examine whether SGI1 integration is limited to its 1^st^ *attB* site, i.e. the last 18 bp of *thdF*, and whether integration in secondary attachment sites may occur, we constructed a Δ*thdF* deletion mutant of *S*. Typhimurium strain LT2 whose genome sequence is available (GenBank accesion number NC_003197) (Table 1) [15]. We realized mating experiments using SGI1-F carrying *S.* Albany strain 7205.00 as donor strain [16] which harbours different somatic O antigens compared to rifampicin-resistant wild type or Δ*thdF* mutant *S.* Typhimurium LT2 recipient strains. As previously described, SGI1 is not self-transmissible and requires additional conjugative functions provided in *trans* by a helper plasmid [13]. Therefore, we introduced the conjugative helper plasmid R55 in the *S.* Albany SGI1 donor strain 7205.00. In the presence of the donor strain, *S.* Albany 7205.00, harbouring the R55 plasmid, SGI1 transconjugants were obtained using the wild type or Δ*thdF* mutant *S*. Typhimurium LT2 recipient strains. The frequency of transconjugants formation was approximately thirty times reduced in the absence of *thdF* (Table 2). Wild type or Δ*thdF* mutant *S*. Typhimurium LT2 transconjugants showed the antibiotic resistance profile conferred by SGI1-F (ApCmFfSuTcTm) [16]. The serovar of transconjugants (Typhimurium) was also confirmed by somatic O antigens agglutination tests and specific PCRs for the retron sequence downstream the *thdF* gene which has been only described in serovar Typhimurium (data not shown). The presence of SGI1 in transconjugants was also confirmed by a set of PCR mappings of the island (antibiotic resistance gene cluster, SGI1 integrase gene *int*) (data not shown) [16]. Since SGI1 is not able to replicate autonomously, the Δ*thdF* mutant *S*. Typhimurium LT2 transconjugants recovered in these experiments likely carried SGI1 integrated in alternative chromosomal attachment sites.

**Table 1:** Bacterial strains, plasmids, and primers used in this study.

Strains and plasmid	Relevant genotype and resistance profile^a^ or characteristics	Reference or source
***S.*** ** enterica**		
Albany 7205.00	SGI1-F^+^; ApCmFfSuTcTm	[16]
Typhimurium LT2	Sensitive, sequenced genome	[15]
Typhimurium LT2	SGI1^−^; Rif	This study
Typhimurium LT2Δ*thdF*::*kan*	SGI1^−^; RifKm	This study
Plasmids		
IncC R55 (*K. pneumoniae*)	Tra^+^; ApCmFfGmKmSu	[19]
pKD4	Derivative pANTSγ, containing an FRT-flanked kanamycin resistance (*kan*); ApKm	[38]
pKD46	Derivative pINT-ts, λ Red recombinase under control of *P_araB_* promoter; Ap	[38]
Primers^b^		
U7-L12	ACACCTTGAGCAGGGCAAAG	[4]
LJ-R1	AGTTCTAAAGGTTCGTAGTCG	[4]
104-RJ	TGACGAGCTGAAGCGAATTG	[4]
C9-L2	AGCAAGTGTGCGTAATTTGG	[4]
104-D	ACCAGGGCAAAACTACACAG	[4]
RecthdF-F	AGGCGGTCATATGACCGCCTTTTTTTATTGCAACAAAGTTGAGACTAACCGTGTAGGCTGGAGCTGCTTC	This study
RecthdF-R	TTACGGGTTTTGTAGGCCCGGTAAGCATCGTGCCACCGGGCAACACAACGCATATGAATATCCTCCTTAG	This study
Linker1	TAATTACACGTTACGACTTCAGATC	This study
Linker2	GATCTGAAGTCGTAACGTG	This study
RvintLM	TTCTTTATTGTGCTGACGCTCTG	This study
SGI1circ1	AGCAAAATCGTGAGAAGGGA	[13]
SGI1circ2	TGATGAGACACCTGACGAGC	[13]
FwsodB	GAAAAATCTCGCCGCATAAG	This study
RvintSGI1	CCTCACCTTCAACAACTCCG	This study
FwS044	CTACCCAGGAGCCACAATCA	This study
RvpurR	GCCCGTTTCGCTACATCTTT	This study

a abbreviations: Ap, ampicillin; Cm, chloramphenicol; Ff, florfenicol; Gm, gentamicin; Km, kanamycin; Rif, rifampicin; Su, sulphonamides; Tc, tetracyclines; Tm, trimethoprim

b Nucleotide sequences are indicated from 5′ to 3′.

**Table 2:** Effect of the Δ*thdF*::*kan* mutation of *S.* Typhimurium LT2 recipient strain on the SGI1 transfer frequency.

SGI1 donor strain	Conjugative helper plasmid R55	Recipient strain	SGI1 transfer frequency^a^
*S.* Albany 7205.00	−	Wild type *S.* Typhimurium LT2	<10^−9^
*S.* Albany 7205.00	−	Δ*thdF* mutant *S.* Typhimurium LT2	<10^−9^
*S.* Albany 7205.00	+	Wild type *S.* Typhimurium LT2	2.1 10^−4^
*S.* Albany 7205.00	+	Δ*thdF* mutant *S.* Typhimurium LT2	7.6 10^−6^

a the frequency of transfer was caculated by dividing the number of SGI1 transconjugants by the number of SGI1 donor cells. Transfer frequencies correspond to the means of three independent mating experiments.

### SGI1 integrates into a unique secondary integration site

**Figure 1:**
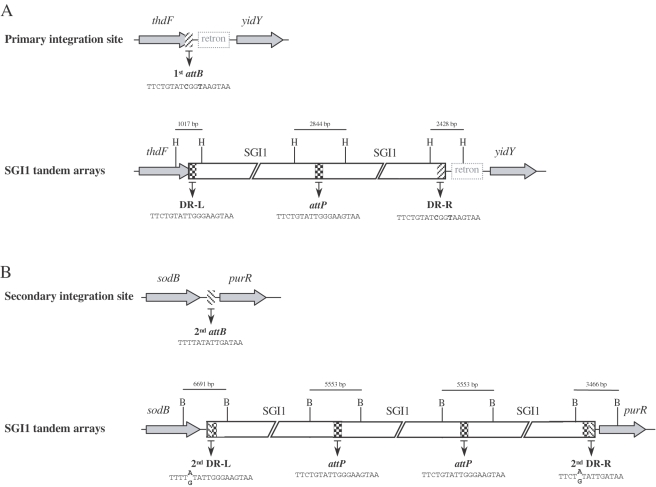
Chromosomal integration sites and tandem arrays of SGI1 in the *S.* Typhimurium chromosome. (A) Schematic view of the primary specific integration site (1^st^ *attB*) of SGI1 within the 3′ end of the chromosomal *thdF* gene of different *S.* Typhimurium strains. The retron sequence downstream the 1^st^ *attB* site is only present in *S.* Typhimurium strains. The integration of SGI1 tandem arrays (2 copies) in its 1^st^ *attB* site is represented. Positions of *Hind*III restriction sites (H) are indicated without respect of bp scale to show the expected sizes of *Hind*III fragments corresponding to 1^st^ DR-L, 1^st^ DR-R, and *attP*. (B) Schematic view of the secondary integration site (2^nd^ *attB*) of SGI1 between the chromosomal *sodB* and *purR* genes. The integration of SGI1 tandem arrays (3 copies) in the 2^nd^ *attB* site is represented. Positions of *Bgl*I restriction sites (B) are indicated without respect of bp scale to show the expected sizes of *Bgl*I fragments corresponding to 2^nd^ DR-L, 2^nd^ DR-R, and *attP*. The sequences of 1^st^ *attB*, 2^nd^ *attB*, *attP*, DR-Ls, and DR-Rs are indicated.

To assess where the integration of SGI1 occurred in the chromosome of the Δ*thdF* mutant *S*. Typhimurium LT2 transconjugants, we examined the left SGI1 junctions in the chromosome for three different transconjugants by ligation-mediated PCR as described in the Materials and Methods section. The SGI1 integration in these transconjugants was determined by sequencing the junctions between the left end of SGI1 and the chromosome. The resulting DNA sequences were then compared to the complete genome sequence of *S.* Typhimurium LT2 (GenBank accession number NC_003197) [15]. Interestingly, by ligation-mediated PCR two PCR products of 550 bp and 900 bp were obtained for each transconjugant tested (data not shown). The sequence of the first one of 550 bp corresponded to the 5′ end of SGI1 linked to the 3′ end separated by the SGI1 *attP* site of 18 bp. This result suggested a potential tandem integration of SGI1 (see below). The sequence of the second 900 bp PCR product corresponded to the 5′ junction in the chromosome. In the three transconjugants, SGI1 was found integrated in the intergenic region between the chromosomal genes *sodB* and *purR* (Fig. 1B). SGI1 was thus integrated downstream of the *sodB* gene coding for the iron superoxide dismutase and 208 bp upstream of the *purR* gene coding for the transcriptional repressor for purine nucleotide synthesis (Fig. 1B). According to the annotated genome sequence of *S.* Typhimurium LT2, the integration of SGI1 would not be predicted to affect the promoter-operator region of *purR*. PCR was performed using primers FwsodB-RvintSGI1 and FwS044-RvpurR corresponding respectively to the left and right junctions of SGI1 integrated between *sodB* and *purR* in the *S.* Typhimurium LT2 chromosome. Ten out of 10 different Δ*thdF* mutant *S*. Typhimurium LT2 SGI1 transconjugants were positive for the left junction between the 5′ end of SGI1 (*int* gene) and the chromosomal *sodB* gene (data not shown). For the right junction, PCR results were positive between the 3′ end of SGI1 (S044) and the *purR* gene of the *S.* Typhimurium LT2 chromosome.

**Figure 2:**
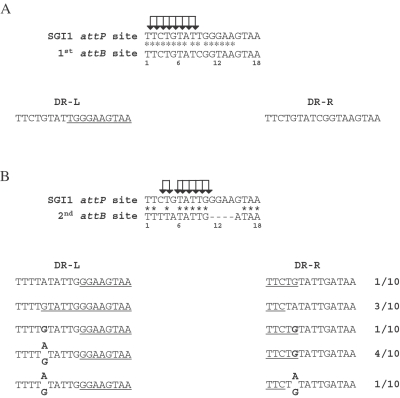
Comparison of the SGI1 18 bp *attP*, *attB*, DR-Ls, DR-Rs of *S.* Typhimurium. (A) Alignment of the *attP* site of SGI1 and the primary *attB* site (1^st^ *attB*) of *S.* Typhimurium strain LT2. The sequence of direct repeats left (DR-L) and right (DR-R) flanking integrated SGI1 were indicated. (B) Alignment of the *attP* site of SGI1 and the secondary *attB* site (2^nd^ *attB*) of *S.* Typhimurium strain LT2. The sequence of direct repeats left (DR-L) and right (DR-R) were determined in ten independent Δ*thdF* mutant *S.* Typhimurium LT2 SGI1 transconjugants from three mating experiments. (*) indicated identical positions in the *attP* site and the *attB* sites. Positions 1, 6, 12, and 18 are indicated below the 1^st^ and 2^nd^ *attB* sequences. The specific nucleotides for *attP* are underlined in DR-Ls and DR-Rs. Sites of possible cleavage during the strand exchange are indicated by arrows in *attP*. Nucleotides in boldface letters represent illegitimate base pair in the DR-L and DR-R sequences. The sequences of DR-L and DR-R of some transconjugants revealed a mix of G and A at position 5 in repeated attempts.

In previous studies, the left and right junctions of SGI1 integrated in the last 18 bp of the *thdF* gene (named 1^st^ *attB* site in this study) have been sequenced and analyzed [4], [13], [16]. The sequence of the specific recombinational site (SGI1 *attP* site) of the extrachromosomal circular form of SGI1 has been also previously determined [13]. Integration of SGI1 in its 1^st^ *attB* site was shown to occur by recombination mediated by the SGI1 integrase between the 18 bp *attP* site of the circular form and the 18 bp 1^st^ *attB* site at the 3′ end of the *thdF* gene [13]. Compared to the SGI1 *attP* sequence, the 1^st^ *attB* site of *S.* Typhimurium strain LT2 presents two nucleotide substitutions at positions 9 and 12 (Fig. 2A). Analysis of DR-L and DR-R in *S.* Typhimurium DT104 in which SGI1 is integrated in its 1^st^ *attB* site demonstrated that these two nucleotide substitutions were always found in the DR-R (Fig. 2A). This result suggests that the cleavage and strand exchange occur somewhere upstream the position 9 during SGI1 integration in its 1^st^ *attB* site (Fig. 2A). The integration of SGI1 in the 2^nd^ *attB* site was slightly different. For the 10 Δ*thdF* mutant *S*. Typhimurium LT2 transconjugants, the sequences of the left and right junctions were determined to analyze the direct repeat sequences flanking SGI1 in this 2^nd^ *attB* site (Fig. 2B). As shown in Figure 2B, the sequence of the 2^nd^ *attB* site in the *S.* Typhimurium LT2 chromosome differs both in length and sequence from the specific SGI1 *attP* sequence. Compared to the SGI1 *attP* site, the 2^nd^ *attB* site is only 14 bp in length and presents three additional substitutions at positions 3, 5, and 15 to the four gap positions (Fig. 2B). The differences in the SGI1 *attP* site and the 2^nd^ *attB* site result in different DR-L and DR-R sequences that allow the cleavage sites during recombination between *attP* and *attB* to be estimated (Fig. 2B). The sequences of DR-L and DR-R suggest that one cleavage and DNA strand exchange occur between bases 3 and 5 (3 out of 10) of the core sequence and the other cleavage and strand exchange occur somewhere between bases 5 and 11 (1 out of 10) (Fig. 2B). Interestingly, in one transconjugant a G nucleotide was found at position 5 in both DR-L and DR-R. In five other transconjugants, a mix of A and G nucleotides was found at position 5 in DR-L or both in DR-L and DR-R (Fig. 2B). The finding of the same base (G) at position 5 in both DR-L and DR-R could be consistent with mismatch repair of single bp substitutions during recombination. Such event has been previously described for the lambda bacteriophage [17]. Furthermore, the mix of bases (A or G) at position 5 in DR-L or both in DR-L and DR-R suggests that in one transconjugant different subpopulations may present different DR-Ls or different DR-Ls and DR-Rs. This finding is consistent with the hypothesis of tandem integration of SGI1 and then different excision events with cleavage and strand exchange upstream or downstream position 5. Thus, SGI1 excision events in tandem arrays could generate for one transconjugant different subpopulations with different DR-Ls or different DR-Ls and DR-Rs.

### Transfer of SGI1 promotes SGI1 tandem arrays in recipient strains

**Figure 3:**
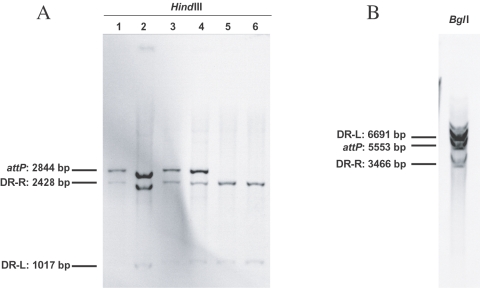
Chromosomal junctions of tandemly-arranged SGI1 islands in the *S.* Typhimurium LT2 chromosome. (A) Southern blot hybridization with the 364-bp *attP* SGI1 probe containing part of S044, 18 bp *attP* site and part of the *int* gene of *Hind*III-digested genomic DNAs of wild type *S.* Typhimurium LT2 SGI1 transconjugants. Lanes 1-4 correspond to different transconjugants with tandem arrays of SGI1 integrated at the 3′ end of *thdF* and lanes 5-6 to transconjugants with a single SGI1 integrated at this *attB* site. (B) Southern blot hybridization with the 364-bp *attP* SGI1 probe of *Bgl*I-digested genomic DNA of Δ*thdF* mutant *S.* Typhimurium LT2 SGI1 transconjugants. All transconjugants tested showed the same profile with *Bgl*I fragments containing DR-L, DR-R, and *attP.* The molecular sizes of *Hind*III or *Bgl*I fragments containing DR-L, DR-R, and *attP* are indicated.

**Figure 4:**
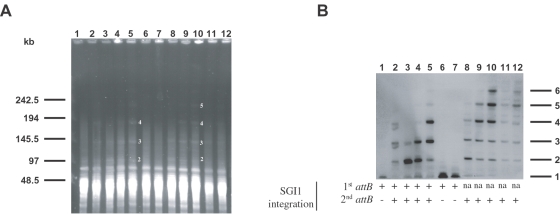
Copy numbers of SGI1 tandem arrays in the *S.* Typhimurium LT2 chromosome. (A) Macrorestriction analysis by PFGE of genomic DNAs cut by *Asc*I of *S.* Typhimurium LT2 SGI1 transconjugants. Lanes: 1, *S.* Albany strain 7205.00 (SGI1 donor strain); 2-7, *S.* Typhimurium LT2 SGI1 transconjugants; 8-12, Δ*thdF* mutant *S.* Typhimurium LT2 SGI1 transconjugants. The molecular sizes in kilobases are indicated to the left of the panel. The numbers indicated to the right of the bands on the restriction patterns (lane 5 and 10) correspond to the copy numbers of SGI1 in each fragment. The size of the *Asc*I fragments observed are consistent with the expected sizes of 2 to 5 SGI1 copies in tandem. (B) Southern blot hybrization with the p1-9 probe of the PFGE-*Asc*I restriction patterns of Figure 4A. The numbers indicated to the right of the panel correspond to the copy numbers of SGI1 in each fragment revealed by the p1-9 probe. The integration sites of SGI1 are indicated under each transconjugant profiles; (+) integrated SGI1, (−) unoccupied *attB* site, (na) not applicable.

To assess whether SGI1 integration occurred in tandem arrays in *S.* Typhimurium LT2 recipient strains, we examined the SGI1 junctions for wild type and Δ*thdF* mutant *S*. Typhimurium LT2 transconjugants by Southern blot hybridization. A 364-bp SGI1 *attP* probe containing part of S044, the 18 bp *attP* site and part of the *int* gene was used. The whole genomic DNAs of 6 wild type *S*. Typhimurium LT2 transconjugants digested by *Hind*III were probed with this SGI1 *attP* probe (Fig. 3A). For all transconjugants, this probe revealed two *Hind*III fragments (Fig. 3A) of the expected sizes (Fig. 1A) corresponding to the left and right junctions when SGI1 is integrated in its 1^st^ *attB* site, i.e., the last 18bp of *thdF*. Four of 6 of the transconjugants studied had a 2.8-kb *Hind*III SGI1 *attP*-specific fragment which corresponded to the link between the 3′end and 5′end of SGI1 (Figs. 1A, 3A). Six Δ*thdF* mutant *S*. Typhimurium LT2 transconjugants were also studied by Southern blot hybridization using this probe and the restriction enzyme *Bgl*I according to the sequence surrounding the 2^nd^ *attB* site (Figs. 1B, 3B). The *attP* probe revealed three *Bgl*I fragments of the expected sizes corresponding to 2^nd^ DR-L, 2^nd^ DR-R, and the 5.5 kb *Bgl*I *attP*-specific fragment (Figs. 1B, 3B). The specific-*attP* fragment revealed in Figure 3A and 3B could be derived from circular extrachromosomal SGI1 or from chromosomal integrated tandem arrays of SGI1. The first possibility appeared unlikely, as in different *Salmonella* field strains carrying SGI1, we were unable to extract a circular intermediate of SGI1 by different alkaline lysis extraction methods and moreover the detection of this circular form by PCR required a nested PCR [13]. Therefore, we used pulsed-field gel electrophoresis (PFGE) to assess whether the specific *attP* fragment revealed by Southern blot hybridization represented integrated copies of SGI1 arranged in tandem or not. To demonstrate SGI1 tandem arrays in PFGE, we used the restriction enzyme *Asc*I which does not cut within SGI1 and frequently cut the *S.* Typhimurium LT2 chromosome in small fragments (around 10 kb in size). According to the genome sequence of *S.* Typhimurium LT2 and the 42,433 bp size of SGI1, the expected sizes of one SGI1 copy integrated at its 1^st^ or 2^nd^ *attB* sites are 51 and 56 kb, respectively. In this manner, the expected sizes of different tandem arrays were determined. DNA from *S.* Albany strain 7205.00, which contained a single SGI1 copy was used as control and 6 wild type and 5 Δ*thdF* mutant *S*. Typhimurium LT2 transconjugants were tested. Compared to the *Asc*I restriction patterns of *S.* Albany strain 7205.00, new bands of higher molecular weight appeared in both the *Asc*I digested DNAs of wild type and Δ*thdF* mutant *S*. Typhimurium LT2 transconjugants (Fig. 4A). To conclude on the copy number of SGI1 arranged in tandem and to exclude the possibility of large *Asc*I chromosomal fragments, we hybridized the PFGE gel with a specific SGI1 probe (p1-9 probe [6]) (Fig. 4B). This Southern blot hybridization revealed six different large fragments of expected sizes consistent with the presence of one, two, three, four, five, and six SGI1s integrated in tandem in the chromosome. The *S.* Albany control strain and 2 out of 6 wild type transconjugants presented a single integrated SGI1 copy (Fig. 4B). For these transconjugants, the integration of SGI1 in its 1^st^ *attB* site and its absence in its 2^nd^ *attB* site was confirmed by PCRs using primers U7L12-LJR1 and FwsodB-RvpurR, respectively (Table 1, Fig. 4B). Interestingly, different SGI1 copy numbers in tandem arrays were found for the four remaining wild type transconjugants and the six Δ*thdF* mutant *S*. Typhimurium LT2 transconjugants. Thus, these results indicated that different subpopulations resulting from a single transconjugant colony contained different copy number tandem arrays of SGI1. These results are in accordance with the given hypothesis on DR-L and DR-R sequence analysis of Δ*thdF* mutant *S*. Typhimurium LT2 transconjugants (Fig. 2B). All these transconjugants were tested for the left and right junctions of SGI1 with the chromosome by PCR to confirm the integration site of SGI1 (Fig. 4B). The 6 Δ*thdF* mutant *S*. Typhimurium LT2 transconjugants were positive for the SGI1 integration in its secondary *attB* site and harboured SGI1 tandem arrays. Interestingly, the 4 wild type transconjugants harbouring SGI1 tandem arrays were positive for integration both in the 1^st^ and 2^nd^ *attB* sites. These results indicated that SGI1 was able to integrate in two distinct *attB* sites in a single wild type *S.* Typhimurium LT2 transconjugant. Thus, the great heterogeneity in subpopulations of SGI1 transconjugants seemed to concern the copy number of tandemly arranged SGI1 and also the integration site.

### Simultaneous integration in two specific chromosomal sites and stability of SGI1 tandem arrays

To study whether integration of SGI1 in wild type *S*. Typhimurium LT2 transconjugants always occurred in the 1^st^ *attB* site, or if SGI1 tandem array formation was correlated with one or both *attB* sites, we further studied the formation of SGI1 tandem arrays and the integration *attB* sites using PCR. One hundred SGI1 transconjugants from three independent mating experiments for the wild type and Δ*thdF* mutant *S*. Typhimurium LT2 recipient strains were tested by PCR junctions for the 1^st^ and 2^nd^ *attB* sites and by PCR for tandem arrays (junction between two copies of SGI1). The frequencies of site integration and tandem array formation are indicated as percent in Table 3. For all wild type *S.* Typhimurium LT2 transconjugants, SGI1 was found integrated in its 1^st^ *attB* site. Forty-three percent of these conjugants also possessed SGI1 integrated in the 2^nd^ *attB* site. Interestingly, the same forty-three transconjugants were positive for SGI1 tandem arrays (Table 3). For the one hundred Δ*thdF* mutant *S*. Typhimurium LT2 transconjugants, they were all positive for integration in the 2^nd^ *attB* site and tandem array formation (Table 3). These results indicated that the SGI1 integration occurred preferentially in its 1^st^ *attB* site. However, approximately half of transconjugants harboured integrated SGI1 copies in the two specific *attB* sites. Moreover, the integration of SGI1 in both *attB* sites seemed to be correlated to tandem array formation. Interestingly, the formation of SGI1 tandem arrays always occurred into its 2^nd^ preferential *attB* site in Δ*thdF* mutant *S*. Typhimurium LT2 transconjugants (in absence of the 1^st^ *attB* site).

**Table 3:** Integration sites and tandem arrays of SGI1 in *S.* Typhimurium strain LT2.

*S.* Typhimurium LT2 transconjugant genotype	Integration in *attB* sites (%)^a^		SGI1 tandem arrays (%)^c^
	Primary site 3′ end *thdF*	Secondary site *sodB*-*purR*	
Wild type *S.* Typhimurium LT2	100	43	43
Δ*thdF* mutant *S.* Typhimurium LT2	na^b^	100	100

a The percent of integration at each site was determined by PCR junctions with the chromosome on one hundred wild type or Δ*thdF* mutant *S.* Typhimurium LT2 transconjugants from three independent mating experiments.

b Not Applicable.

c The percent of SGI1 tandem arrays was determined by PCR using primers SGI1circ1 and SGI1circ2.

The SXT element from *Vibrio cholerae* was also able to integrate in a tandem fashion in *E. coli* into its specific integration site [18]. However, after 5 days cultures only one copy number of SXT was detected in the *E. coli* transconjugant suggesting a decrease from a multiple copy number arranged in tandem to only one after this time [18]. To investigate the stability of SGI1 tandem arrays in *S.* Typhimurium LT2, wild type and Δ*thdF* mutant transconjugants were cultivated for 15 days with two dilutions per day in fresh medium (approximately >600 generations) with or without antibiotic selection for SGI1. Wild type transconjugants with only one copy of SGI1 or with tandem arrays were included in this experiment. Throughout this time, bacterial cultures were tested by PCR for SGI1 tandem arrays and at the end time for integration into *attB* sites. With or without antibiotic selection, no changes were observed by PCRs for integration sites, single SGI1 copy or tandem arrays during this time (data not shown). Unlike the *Vibrio cholerae* SXT element, SGI1 tandem arrays appeared to persist after several cultures with or without antibiotic selection with at least two SGI1 copies arranged in tandem.

## Discussion

*Salmonella* genomic island 1 (SGI1) is an integrative mobilizable element (IME) containing an antibiotic resistance gene cluster identified in several *S. enterica* serovars and recently also in *P. mirabilis* [5], [6], [8], [10], [16]. In a previous study, SGI1 was found to transfer by conjugative mobilization, using conjugative helper plasmid R55 [19], from a *S. enterica* donor to a recipient strain (*E. coli* or *S. enterica*) [13]. In the donor strain, the excision and circularization of SGI1 is mediated by the SGI1-encoded integrase Int which presents similarity to the λ integrase family (Tyrosine recombinase family) [13], [20]. The Int-mediated recombination between the 18 bp direct repeats left and right (DR-L and DR-R) flanking the integrated SGI1 results in a unique 18 bp sequence (*attP* site) in the SGI1 circular form. The SGI1 integration into the chromosome of the recipient occurs by recombination between the SGI1 *attP* site of the circular form and the chromosomal 1^st^ *attB* site, i.e., the last 18 bp of the *thdF* gene [13]. The site-specific integration of SGI1 in the chromosome demonstrated experimentally, is also supported by the growing number of *S. enterica* serovars and *P. mirabilis* strains in which SGI1 was found to be integrated at the 3′ end of the chromosomal *thdF* gene [5], [8]–[10]. Thus, SGI1 represents a non replicative element which needs to integrate in the chromosome to persist in the host strain [4], [6], [13]. In this study, in absence of the *thdF* gene, SGI1 was found to integrate in a specific 2^nd^ *attB* site between the chromosomal *sodB* and *purR* genes. However, in some transconjugants containing *thdF*, SGI1 was found integrated in the two attachment sites, *thdF* and *sodB*-*purR,* with at least one copy in each attachment site. Moreover, tandem arrays of SGI1 were always found in *S.* Typhimurium LT2 SGI1 transconjugants lacking *thdF*. There was heterogeneity in the size of SGI1 tandem arrays detected in cells from single transconjugants. Tandem arrays contained different copy numbers of SGI1 ranging in size from two to six repeats.

Various elements, including phages, integrative conjugative elements and pathogenicity islands have been described to integrate site-specifically in one site and also in secondary attachment sites [18], [21]–[26]. Other mobile elements such as the SRL PAI of *Shigella flexneri*, the clc element of *Pseudomonas* sp. strain B13, and the SXT element of *V. cholerae* share with SGI1 very similar properties of integration [3], [22], [26]–[28]. The 66-kb SRL (*Shigella* resistance locus) PAI (pathogenicity island) in *Shigella* spp. mediates multiple antibiotic resistances and integrates site-specifically into two bacterial tRNA *attB* sites [22], [27]. The integrase Int of SRL PAI mediates the integration adjacent to one or both identical paralogous tRNA genes *serX* and *serW* [22], [27]. Chromosomal integrations of the 105-kb clc element of *Pseudomonas* sp. strain B13 occurred also in two similar sites which are the glycine tRNA genes in the *Pseudomonas* chromosome [28]. The SRL PAI island and the clc element are able to integrate in one or both identical *attB* sites of the host chromosome. The SXT element of *V*. *Cholerae* is a conjugative self-transmissible chromosomally integrating element which also contains several antibiotic resistance genes [3], [29]. SXT integrates site-specifically at the 5′ end of the chromosomal *prfC* gene [26]. In the absence of *prfC*, the SXT element integrates in several secondary attachment sites but preferentially into the 5′ end of the chromosomal *pntB* gene [26]. Moreover, the SXT element is also able to integrate in a tandem fashion after conjugative transfer [18].

The mechanism accounting for the formation of SGI1 tandem arrays is unknown but this event is likely related to the conjugative transfer of SGI1. To date in *S. enterica* field strains harbouring SGI1, we have never detected SGI1 arrays (data not shown). Serial passage of representative field strains containing a single SGI1 did not result in amplification of the SGI1 copy number (data not shown). Several conjugative-dependent mechanisms could explain the formation of tandem SGI1 arrays. Tandem arrays could form if a concatemer of several SGI1 copies was transferred from a single donor cell to a single recipient cell. SGI1 being a mobilizable element, its conjugative transfer is similar to conjugative plasmids transfer. We hypothesize that a single-stranded SGI1 generated by a rolling circle process, is transferred from donor to recipient. The general model of bacterial conjugation proposes that a single strand is transmitted in the 5′ to 3′ orientation to the recipient cell [30]. This transfer process is initiated by nicking DNA and finished by religation at the origin of transfer (*oriT*) resulting in a monomeric circle of transferred DNA. During the transfer, synthesis of the replacement strand by a rolling circle mode of DNA replication reconstitutes the transferred single strand [30]. Thus, concatemer of several copies could be transferred to the recipient. Alternatively, a single donor may transfer a single SGI1 but in repeated attempts to a single recipient cell. Another explanation is that a single recipient cell could be implicated in successive conjugation events with different donor cells and thus acquired several SGI1 copies.

Interestingly, we demonstrated that the SGI1 integration in the 2^nd^ *attB* site always occurred in a tandem fashion in absence of the 1^st^ *attB* site (Table 3). This result is consistent with a SGI1 concatemer integration in the 2^nd^ *attB* site. Moreover, according to the lower frequency of transfer in the Δ*thdF* mutant *S.* Typhimurium LT2 recipient strains, this hypothesis could be more probable than successive transfers of single SGI1 monomer into a single recipient cell. However, the formation of SGI1 tandem arrays also occurred in the wild type *S.* Typhimurium LT2 recipient strain. Fifty-seven of one hundred transconjugants tested, presented a single SGI1 integrated in the 1^st^ *attB* site (Table 3, Fig. 4B). In contrast, for fourty-three transconjugants positive for tandem arrays, integration of SGI1 occurred into the two *attB* sites (Table 3). This result suggests that tandem array formation could occur in both *attB* sites. Thus, some wild type transconjugants may contain SGI1 tandem arrays integrated in the 1^st^ *attB* site (Figs. 3, 4) and one or several SGI1 copies integrated in the 2^nd^ *attB* site. DNA fingerprint analysis using PFGE in Figure 4 of wild type *S.* Typhimurium LT2 transconjugants did not permit a conclusion as to where SGI1 tandem arrays were integrated between the 1^st^ and 2^nd^ *attB* sites (only 5 kb size difference). However, the absence of detectable hybridized fragments corresponding to the 2^nd^ DR-L and 2^nd^ DR-R in Southern blot hybridization using the SGI1 *attP* probe (Fig. 3A) for the four transconjugants containing tandem arrays suggested that the SGI1 tandem arrays were integrated in the 1^st^ *attB* site.

Several questions remain to be answered: (i) were there two independent conjugative transfers resulting in the occupancy of the two *attB* sites; or (ii) was there in a first time a SGI1 concatemer transfer and integration in the 1^st^ *attB* site and then excision of one or several SGI1 copies and reintegration in the 2^nd^ *attB* site in a subpopulation of a single transconjugant. To assess these hypotheses, further studies need to be undertaken to demonstrate a potential sequential event. For the *stx*_2_ bacteriophages of Shiga toxin-producing *E. coli*, the insertion site occupancy by stx phages depends on the availability of the preferred site in the host strain [21]. If the primary insertion site is unavailable, then a secondary insertion site is selected [21]. In contrast, the integrative conjugative element SXT from *V. cholerae* is able to use the left or right direct repeats of a previously integrated element for integration [31].

The analysis of SGI1 copy number in tandem arrays in transconjugants revealed considerable heterogeneity (Fig. 4). Although formation of tandem SGI1 arrays appears to occur frequently in *S.* Typhimurium LT2 chromosome after conjugative transfer *in vitro*, these arrays probably decreased in size during bacterial multiplication resulting in different subpopulations with different SGI1 copy numbers in a single colony. Homologous recombination between SGI1 copies in a tandem array may lead to a decreased SGI1 copy number in arrays. Another explanation is that the SGI1 integrase Int could excise single or multiple SGI1s by recombination involving the DR-L and an internal *attP* site of the tandem arrays, or DR-R and an internal *attP* site, or two internal *attP* sites. All these recombinations would result in the formation of circular extrachromosomal forms containing a single or several SGI1s in the bacteria. According to the previous speculation, such circular intermediates could be implicated in integration in the remaining *attB* site. Moreover, analysis of DR-L and DR-R in Δ*thdF* mutant *S.* Typhimurium LT2 transconjugants (Fig. 2B) demonstrated different DR-L and/or DR-R sequences in some transconjugants with mix of bases at position 5. This result suggested that integration or excision of SGI1 copies occurred at the left and/or right side of tandem arrays in the 2^nd^ *attB* site.

**Figure 5:**
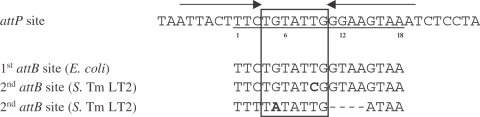
Analysis of the SGI1 *attP* overlapping region. The previously described 18-bp sequence of the *attP* site is underlined. Positions 1, 6, 12, and 18 are indicated below the *attP* site. The imperfect inverted repeats are indicated by arrows. Sequences of the different SGI1 *attB* sites are aligned below the SGI1 *attP* sequence. Nucleotide in boldface letters represented substitutions compared to the *attP* sequence. The black box represented the putative 7-bp overlap region in which cleavages and strand exchanges occurred.

The properties of site-specific integration of SGI1 appear very similar to those of the λ integrase family [20]. Analysis of the SGI1 *attP* region using DNA strider 1.4f3 software and the Mfold web server revealed two 8-bp imperfect inverted repeats surrounding and partially within the 18-bp sequence of the SGI1 *attP* site (Fig. 5) [32]. These imperfect inverted repeats could correspond to the integrase inverted-core binding sites suggesting that the overlap region in which cleavage and strand exchange occurred, could be restricted to a 7-bp central overlap region. Interestingly, several mobile elements such as λ, CTnBST, CTnDOT, NBU1 also have 7 bp between the cleavage sites within inverted repeat sequences in their respective *attP* sites [20], [25], [33], [34]. Moreover, this 7-bp overlap region is in accordance with the potential cleavage sites estimated in Figure 2. The integrase of SGI1 (IntSGI1) has been previously described as a member of the λ integrase family (Tyrosine recombinase family) because IntSGI1 has five of six highly conserved residues found in the catalytic domains of this family of recombinases [13]. Interestingly, Figure 5 shows that different positions located within the 7-bp overlap region of the SGI1 *attB* sites could be substituted. In the SGI1 1^st^ *attB* site of *S.* Typhimurium strains, position 9 is a C, while in SGI1 *attP* site has a T (Fig. 2A). In the 2^nd^ *attB* site, it is position 5 which is substituted (Fig. 2B). Interestingly, in several other *S. enterica* serovars previously described to harbour SGI1 integrated in its 1^st^ *attB* site and also in *E. coli* previously used as recipient for *in vitro* SGI1 transfer, no substitution was observed in the 7-bp overlap region in unoccupied *attB* sites [6], [13], [16]. For λ integration, the overlap regions of the *attP* and *attB* sites must be perfectly homologous for efficient recombination to occur [20]. In contrast, the integration of the *Bacteroides* CTnBST element requires homology at only one end of the crossover region but not at the other end [25]. According to the strand-swapping model proposed for λ system, a Holliday junction is formed following two symetrical swaps of two or three nucleotides resulting in a branch located near the center of the 7-bp overlap region. After an isomerization step from one strand crossover to the other strand crossover, the second strand swap resolves the Holliday junction [20]. Studies of the homology-dependent steps during integrative recombination of λ demonstrate that the first-strand cleavage is strongly dependent on the presence of homology between the first 3 bp of overlap regions [20], [35]. The lower transfer frequency using Δ*thdF* mutant *S*. Typhimurium LT2 recipient compared to wild type could be an indication for a lower integration frequency in the 2^nd^ *attB* site potentially due to the substitution at the position 5 in the 7-bp overlap region (Fig. 5). However, other integrase-binding sites like arm type sites or core-type sites are also described to play an important role in integration frequency [20]. Site-directed mutagenesis could be used to establish which positions within this putative 7-bp overlap region of *attB* sites are critical for the integration of SGI1.

In summary, we have shown that the genomic island SGI1 is able to integrate in a secondary *attB* site which is highly conserved amongst the different *Salmonella* sequenced genomes (data not shown). After conjugative transfer, SGI1 tandem arrays are integrated in both *attB* sites with a great heterogeneity in the size of the tandem arrays in single transconjugant colonies. The ability of integration into distinct chromosomal sites could contribute to the spread and persistence of SGI1. Thus, SGI1 could possibly integrate in other bacterial pathogens that do not possess either the 1^st^ or 2^nd^ SGI1 *attB* sites but a slightly divergent *attB* site. It is interesting to note that several genomic islands implicated in multidrug resistance are now described to use site-specific integration in the host chromosome as a mean for persistence after horizontal transfer. This study provides an interesting insight into potential mechanisms that strengthen the spread of multiple antibiotic resistance among human bacterial pathogens.

## Materials and Methods

### Bacterial strains, plasmids and antibiotic susceptibility testing

The *Salmonella* strains used in conjugation experiments are described in Table 1. *S.* Albany strain 7205.00 harbouring the SGI1-F variant was used as donor strain [16]. *S.* Typhimurium strain LT2 was made rifampicin resistant as previously described [15], [36]. All strains were grown at 37°C in brain heart infusion broth or agar plates. IncC conjugative plasmid R55 from *Klebsiella pneumoniae* was used as a helper plasmid for mobilization experiments as previously described [13], [19]. Donor, recipient, and transconjugant strains were screened for antibiotic resistance by the disk diffusion method on Mueller-Hinton agar plates [37]. Susceptibility was tested using disks containing the following antibiotics: Ap (10 μg), Cm (30 μg), Ff (30 μg), Km (30 IU), Gm (15 μg), Sm (10 IU), Sp (100 μg), Su (200 μg), Tc (30 IU) and trimethoprim (Tm) (5 μg). All antibiotic disks except Ff were purchased from BioRad (Marnes la Coquette, France). Ff disks were obtained from Schering-Plough Animal Health (Segré, France).

### Deletion of the *thdF* gene by insertion mutagenesis

Deletion of the chromosomal *thdF* gene was performed in *S.* Typhimurium strain LT2 using the one step chromosomal gene inactivation technique [38]. Briefly, the kanamycin resistance gene *kan* flanked by FRT (FLP recognition target) sites was amplified by standard PCR using the template plasmid pKD4 and hybrid primers. These primers, RecthdF-F and RecthdF-R (Table 1), were synthesized with 20 nucleotides of priming sites of pKD4 and with 50 nucleotides from each side of the *thdF* gene. The 1.6 kb long PCR fragment was purified and electroporated into the *S.* Typhimurium strain LT2 in which the λ Red recombinase expression plasmid pKD46 was introduced. Homologous recombination between the genomic DNA and the PCR product resulted in the deletion of the entire *thdF* gene and in its replacement with the *kan* gene. The resulting strain was named Δ*thdF* mutant *S.* Typhimurium LT2 compared to the wild type *S.* Typhimurium strain LT2.

### Bacterial conjugations

Conjugation assays were performed by mixing *S.* Albany SGI1 donor strain 7205.00 with or without the helper plasmid R55 and the rifampicin resistant *S.* Typhimurium LT2 recipient strains (wild type or Δ*thdF* mutant) together with a donor-to-recipient ratio of 4:1. This broth was incubated overnight at 37°C without shaking. The next day, the cells were streaked on appropriate selective brain heart infusion agar plates. Rifampicin (250 μg/ml) was used to select against *S.* Albany donor cells, and Tc (10 μg/ml) to select against unmated recipient cells. The SGI1 frequency of transfer was determined by dividing the number of SGI1 transconjugants by the number of *S.* Albany SGI1 donor cells. Transconjugants were tested for antibiotic resistance, for somatic O antigens by agglutination tests with antisera (Bio-Rad, Manes la Coquette, France), and also by PCR for specific markers described below.

### Secondary attachment site determination by ligation-mediated PCR

The secondary integration sites of SGI1 were determined by performing ligation-mediated PCR as described below. Genomic DNAs of Δ*thdF* mutant *S.* Typhimurium LT2 SGI1 transconjugants were cut by blunt-end restriction enzymes *Alu*I or *Eco*RV (Promega, Charbonnieres, France). Annealing of the two primers Linker1 and Linker2 to form the double-stranded adaptators was performed by boiling a 5 nM solution of the mixed primers, followed by slow cooling to room temperature. *Alu*I or *Eco*RV digested chromosomal DNAs were ligated to adaptators in 10 µl final volume at a 10-fold molar excess of the adaptator, according to the number of generated fragments.

A first round of amplification was performed by using the primer Linker1 and the first SGI1 internal primer RvintLM to the left end of SGI1 (Table 1), in 25 µl PCR mixtures with a GoTaq Master Mix kit (Promega, Charbonnieres, France) and 2 µl of ligation. The first-round PCR conditions were (i) 5 min at 95°C, (ii) 30 cycles of 30 s at 95°C, 30 s at 60°C, and a variable elongation time at 72°C according to the length of generated fragments, and (iii) 7 min at 72°C. The second round of amplification was performed like the first round with 2 µl of the first-round reaction mixture as the template and primers Linker1 and LJR1, which was identical to the leftmost end of SGI1 (Table 1). The second-round amplification conditions were (i) 5 min at 95°C, (ii) 30 cycles of 30 s at 95°C, 30 s at 57°C, and variable elongation time at 72°C, and (iii) 7 min at 72°C. The purified PCR products were sequenced by using the SGI1 LJR1 primer at Genome Express (Meylan, France) and were compared with the GenBank DNA sequence database by using the genomic BLASTN program.

### PCR mapping, sequencing, Southern blot hybridization

Detection of SGI1 and its location were performed using primers corresponding to the left and right junction in the 1^st^ and 2^nd^ *attB* integration site (Table 1, Fig. 1). PCR products corresponding to the left and right junctions at the secondary *attB* site of ten independent Δ*thdF* mutant *S.* Typhimurium LT2 transconjugants were sequenced. Nucleotide sequencing was achieved by Genome Express (Meylan, France). For the wild type and Δ*thdF* mutant *S.* Typhimurium LT2 recipient strains, one hundred independent transconjugants of three different mating experiments were screened by PCR on the left junction for SGI1 integration (1^st^ and 2^nd^ *attB* sites) and by PCR using primers SGI1circ1 and SGI1circ2 oriented towards the left and right end of SGI1 for tandem integration (Table 1, Fig. 1).

To assess tandem arrays of SGI1, Southern blot analysis of wild type and Δ*thdF* mutant *S.* Typhimurium LT2 transconjugants was performed. Briefly, total genomic DNAs of transconjugants were digested with *Hind*III or *Bgl*I and hybridized with the 364-bp amplified fragment containing part of S044, *attP*, and part of SGI1 as a probe. The expected sizes of fragments containing DR-L, DR-R, and *attP* in wild type and Δ*thdF*::*kan* transconjugants correspond to 1017, 2428, 2844 bp *Hind*III fragments and 6691, 3466, 5513 bp *Bgl*I fragments, respectively.

### Copy number of SGI1 in tandem arrays

Chromosomal DNA of wild type and Δ*thdF* mutant *S.* Typhimurium LT2 SGI1 transconjugants strains was prepared for pulsed field gel electrophoresis as previously described [16]. Genomic DNA was digested with *Asc*I restriction enzyme (BioLabs, Saint Quentin, France), which do not cut within SGI1 but relatively frequently in the chromosome of *S.* Typhimurium LT2. Fragments of DNA were separated by pulsed-field gel electrophoresis (PFGE) in a 1% agarose gel (BioRad, Marnes la Coquette, France) by using a CHEF-DR III (Bio-Rad, Hemel Hempstead, United Kingdom). The running conditions were 6 V/cm at 14°C for 22 h, with pulse times ramped from 7 to 20 s. Southern blot hybridization was realized on *Asc*I PFGE using the p1-9 probe previously described to assess the copy number of SGI1 in tandem arrays.
